# Prognostic impact of tumour-associated B cells and plasma cells in epithelial ovarian cancer

**DOI:** 10.1186/s13048-016-0232-0

**Published:** 2016-04-06

**Authors:** Sebastian Lundgren, Jonna Berntsson, Björn Nodin, Patrick Micke, Karin Jirström

**Affiliations:** Department of Clinical Sciences, Division of Oncology and Pathology, Lund University, SE-221 85 Lund, Sweden; Department of Immunology, Genetics and Pathology, Uppsala University, SE-751 85 Uppsala, Sweden

**Keywords:** Immunoglobulin kappa c, CD20, CD138, Syndecan-1, Ovarian cancer, Prognosis

## Abstract

**Background:**

The critical role of the immune system in controlling cancer progression has become evident and immune modulatory therapy is now approved for clinical use. However, while the majority of studies on the inflammatory tumour microenvironment have focused on the cellular immune response, in particular the prognostic and predictive role of various T cell infiltrates, the role of the humoral immune response in this context has long been overlooked. This study aimed to investigate the clinicopathological correlates and prognostic impact of B cell and plasma cell infiltration in epithelial ovarian cancer (EOC).

**Methods:**

Immunohistochemical expression of immunoglobulin kappa C (IGKC), CD20 and CD138 was analysed in tissue microarrays with tumours from 154 incident cases of EOC from two pooled prospective population-based cohorts. Subsets of corresponding benign-appearing fallopian tubes (*n* = 38) and omental metastases (*n* = 33) were also analysed. Kaplan-Meier analysis and Cox regression analysis were used to determine the impact of immune-cell specific IGKC, CD20 and CD138 expression on overall survival and ovarian cancer-specific survival.

**Results:**

High IGKC expression correlated significantly with expression of CD20 (*p* = 0.001) and CD138 (*p* = 0.035). Expression of IGKC as well as CD138 was significantly higher in primary tumours than in fallopian tubes (*p* = 0.004 and *p* = 0.001, respectively). High CD20 and CD138 expression correlated significantly with high tumour grade (*p* = 0.032 and *p* = 0.030, respectively). CD20 and IGKC expression was not prognostic but univariable Cox regression analysis revealed high CD138 expression to correlate with a significantly reduced overall survival (HR = 2.20; 95 % CI 1.34–3.55; *p*–0.001) as well as ovarian cancer-specific survival (HR = 1.95; 95 % CI 1.28–2.98; *p* = 0.002). The prognostic impact was independent of established clinical parameters (age, grade, clinical stage) as shown in multivariable analysis (HR = 2.28; 95 % CI 1.39–3.75; *p* = 0.001).

**Conclusions:**

In conclusion, our results demonstrate that plasma cell infiltration in epithelial ovarian cancer has a significant impact on tumour progression and prognosis. The important role of the humoral immune system merits further study and may be harnessed as immune modulatory strategies in cancer therapy.

**Electronic supplementary material:**

The online version of this article (doi:10.1186/s13048-016-0232-0) contains supplementary material, which is available to authorized users.

## Background

Cancer immunity has emerged as a clinically relevant hallmark of cancer biology [1]. The immune system plays a multifaceted role and may in different contexts promote or inhibit tumour growth [2]. On the one hand, inflammation caused by cancer and specific immune cell infiltrates have demonstrated tumorigenic properties [3]. On the other hand, inflammatory cells, in particular of the T-cell lineage, are associated with inhibiting properties and are capable to eliminate tumor cells [4]. As a proof of concept, so-called checkpoint inhibitors, modulating the T-cell response, have been shown to impress response rates in different solid tumours and are now approved for the treatment of lung cancer and melanoma [5, 6].

Consequently, the majority of studies concerning the role of the immune system in cancer have focused on the cellular response. Indeed, high infiltration of specific T-lymphocytes has been associated with favourable clinical outcome in many tumour types and an immune index has been suggested to outperform classical prognostic parameters in colon cancer [7]. The humoral anti-tumour response, however, has been far less investigated.

Immunoglobulin kappa C (IGKC), expressed in plasma cells, has in breast cancer been identified as one of the top genes of a prognostic B cell-metagene [8] and is related to favourable prognosis and response to chemotherapy [8]. The auspicious impact of IGKC has been confirmed in non-small cell lung cancer [9] and colorectal cancer [10] and in the former study, CD138-expressing plasma cells were identified as the cellular correlate for the gene expression signature.

Also, in epithelial ovarian cancer (EOC), tumour-specific CD138 expression has been shown to be associated with enhanced cell invasion [11] and poor patient outcome [12, 13]. CD20+ tumour infiltrating lymphocytes (TILs), i.e. mature B cells who have undergone Ig-class switching [14], are reportedly strongly linked to an improved patient outcome in high-grade serous ovarian cancer when combined with CD8+ T cells [15]. However, another study, analysing only IGKC mRNA expression, did not find any correlation with survival [10].

The aim of this study was to examine the immunohistochemical (IHC) expression and clinical correlates of IGKC, CD20 and CD138 protein expression in tumours from 154 EOC cases from two pooled, prospective, population-based cohorts. Furthermore, we aimed to investigate if these markers correlate with expression of the polymeric immunoglobulin receptor (PIGR), which has previously been described as an indicator of a less aggressive tumour phenotype and improved survival in EOC [16].

## Methods

### Patients

The study cohort is a merge of all incident cases of EOC in the two prospective population-based cohorts Malmö Diet and Cancer Study (MDCS) (*n* = 101) [17] and Malmö Preventive Project (MPP) (*n* = 108) [18] until December 31st 2007, as previously described [16, 19–22]. Information on vital status and cause of death was obtained from medical charts and the Swedish Cause-of-Death Registry up until June 30 2012. After a median follow-up of 3.00 years (range 0–24.63), 122 patients (79.2 %) were dead, 112 (72.3 %) from ovarian cancer, and 32 (20.8 %) were alive. All tumours were re-evaluated regarding histological subtype and grade, by a senior pathologist (KJ). Tumours were categorised into four groups according to histological subtype: serous (*n* = 90), endometrioid (*n* = 35), mucinous (*n* = 12) and others (*n* = 17). The group of others included clear cell (*n* = 9), Brenner (*n* = 1) and unknown (*n* = 7) tumours. Histopathological, clinical and treatment data were obtained from clinical and/or pathology records. Information on residual tumour after surgery was not available. Standard adjuvant therapy was platinum-based chemotherapy in combination with paclitaxel.

Ethical permission for the study was approved by the Ethics Committee of Lund University (Ref 445/2007).

### Tissue microarray construction

Tissue microarrays (TMAs) were constructed as previously described [16, 19, 22], whereby two 1.0 mm. cores were taken from viable, non-necrotic primary tumour areas, and from concomitant peritoneal metastases (*n* = 34). Fallopian tubes with no evidence of histological disease were also sampled from 38 cases.

### Immunohistochemical staining and evaluation

For IHC analysis of CD138 and CD20, 4 μm TMA-sections were pre-treated using ULTRA Cell Condition Solution 1, pH 8.5 (Ventana Medical Systems, Tucson, AZ, USA) ULTRA Cell Conditioning (ULTRA CC1), for heat induced epitope retrieval, and stained with the ready-to-use monoclonal antibodies CD20cy Clone L6 and CD138 clone MI15 in a Ventana BenchMark stainer (Ventana Medical Systems Inc.). The antibody-antigen complex was visualized with ultraView Universal DAB Detection kit (Ventana Medical Systems, Inc.).

For analysis of IGKC, the TMA slides were manually deparaffinised in xylene, rehydrated in graded alcohol and blocked for endogenous peroxidase in 0.3 % hydrogen peroxide. For antigen retrieval, the slides were immersed in Citrate Buffer pH 6.7 and microwaved for 15 min. Automated IHC was performed with the Autostainer Link 48 (Dako; Glostrup, Copenhagen, Denmark) and a polyclonal rabbit anti-human kappa light chain antibody (Dako, A019;1:40 000). The slides were incubated with the secondary antibody (EnVision™ FLEX, Rabbit/Mouse, K8000, Dako) for 30 min at RT and developed using diaminobenzidine (DAB). All TMA slides were counterstained with Mayer’s hematoxylin (Sigma–Aldrich).

Evaluation of the IHC staining in immune cells was annotated by two assessors (JB, SL), whereby consensus for each core was reached in estimated percentage as a continuous value. The staining intensity was annotated in categories of 0–2, where 0 = negative, 1 = intermediate and 2 = strong intensity.

Core score (CS), i.e. a multiplier of intensity and fraction, was calculated for each individual core assessed for IGKC, CD20 and CD138 staining and a mean value of the two corresponding cores was used in the statistical analyses.

For CD138, tumour-specific expression was also annotated, whereby an estimated percentage of stained cells was reached in estimated percentage groups as follows; negative (0 %), 1–25 %, 26–50 %, 51–75 % and 76–100 %. Staining intensity was annotated in groups of 0–3, whereby 0 = negative, 1 = weak, 2 = moderate and 3 = strong intensity. A multiplier of intensity and fraction was calculated for each core and a quotient of the multiplier and fraction of tumour cells was calculated and used in the analyses.

Analysis of IHC expression of PIGR had been performed as previously described [16]. KRAS mutation status had been determined by pyrosequencing as previously described [23].

### Statistical analysis

Mann–Whitney U-test was used to assess distribution differences in expression of IGKC, CD20 and CD138 in relation to clinicopathological characteristics and other investigative biomarkers. Spearmans Rho test was used to analyse the interrelationship between IGKC, CD20 and CD138. Paired T-test was used to illustrate differences of biomarker expression in primary tumours, metastases and fallopian tubes. Classification tree (CRT) was used to evaluate optimal cut off for dichotomisation of biomarker expression. Kaplan-Meier analysis and log rank test were applied to illustrate differences in overall survival (OS) and ovarian cancer specific survival (OCSS) with respect to IGKC, CD20 and CD138 expression. Cox regression proportional hazard models were used to estimate hazard ratios (HRs) for death from EOC or overall causes according to high and low expression of the investigative markers in both uni- and multivariable analysis, adjusted for age, stage and differentiation grade. All calculations were performed using SPSS version 22.0 (SPSS Inc, Chicago, IL). All statistical tests were two-sided and *p-values* < 0.05 were considered statistically significant.

## Results

### Distribution of IGKC, CD20 and CD138 expression in fallopian tubes, EOC and metastases

**Fig. 1:**
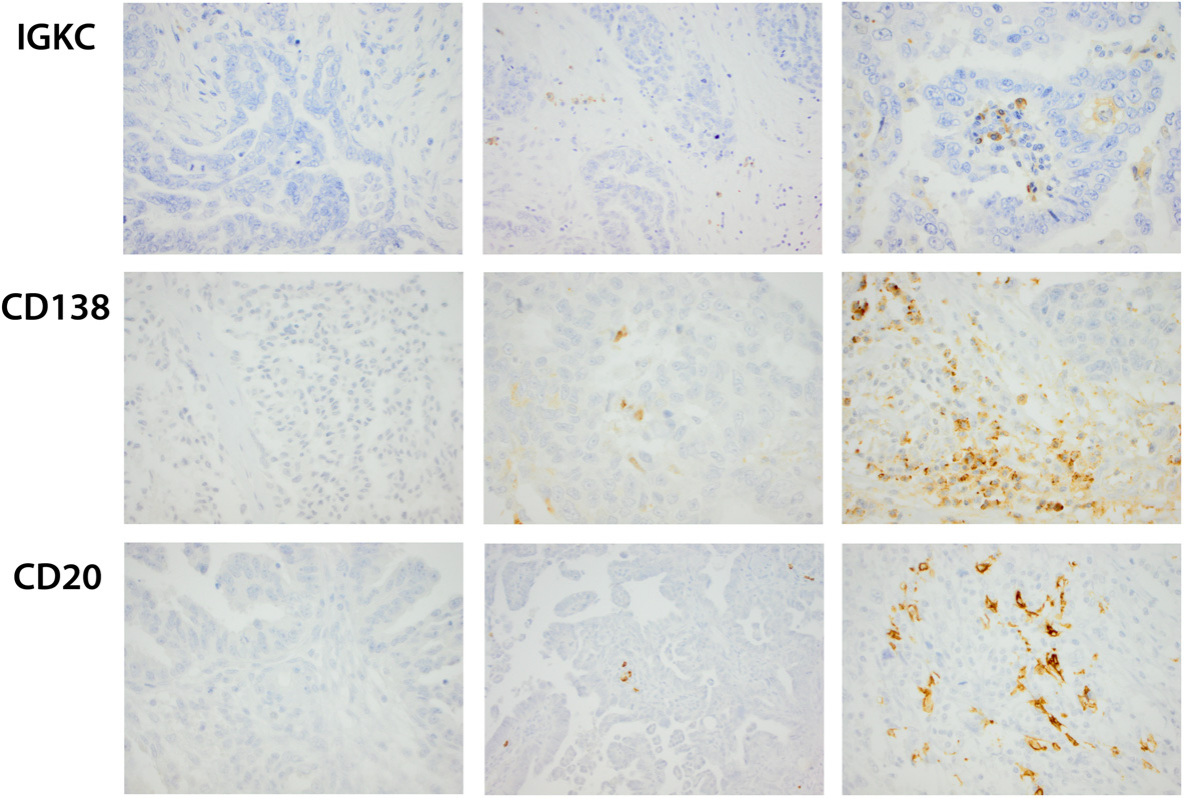
Immunohistochemical images of IGKC, CD20 and CD138 staining in fallopian tubes, primary and metastatic epithelial ovarian cancer. Sample images (40X magnification) representing immunohistochemical expression of IGKC (first row), CD138 (second row) and CD20 (third row), described as core score, i.e. a multiplier of intensity (0–3) and fraction of staining; left column representing negative CS, middle column intermediate CS and right column high CS

Quantification of immunohistochemical expression of IGKC, CD20 and CD138 is illustrated in Fig. 1. IGKC expression was evaluable in 151 (98.1 %) of the primary tumours, 33 (97.1 %) of the metastases and in 32 (82.4 %) of the corresponding fallopian tubes. CD20 expression was evaluable in all 154 of the primary tumours, in 33 (97.1 %) of the metastases and in 34 (89.5 %) of the fallopian tubes. Finally, immune cell-specific CD138 expression could be assessed in 151/154 (98.1 %) samples from primary tumours, in all 34 metastases and in 34/38 (89.5 %) samples from fallopian tubes, whereas tumour-specific expression of CD138 was quantifiable in 151 (98.1 %) of the primary tumours and in 30 (88.2 %) of the metastases.

**Fig. 2:**
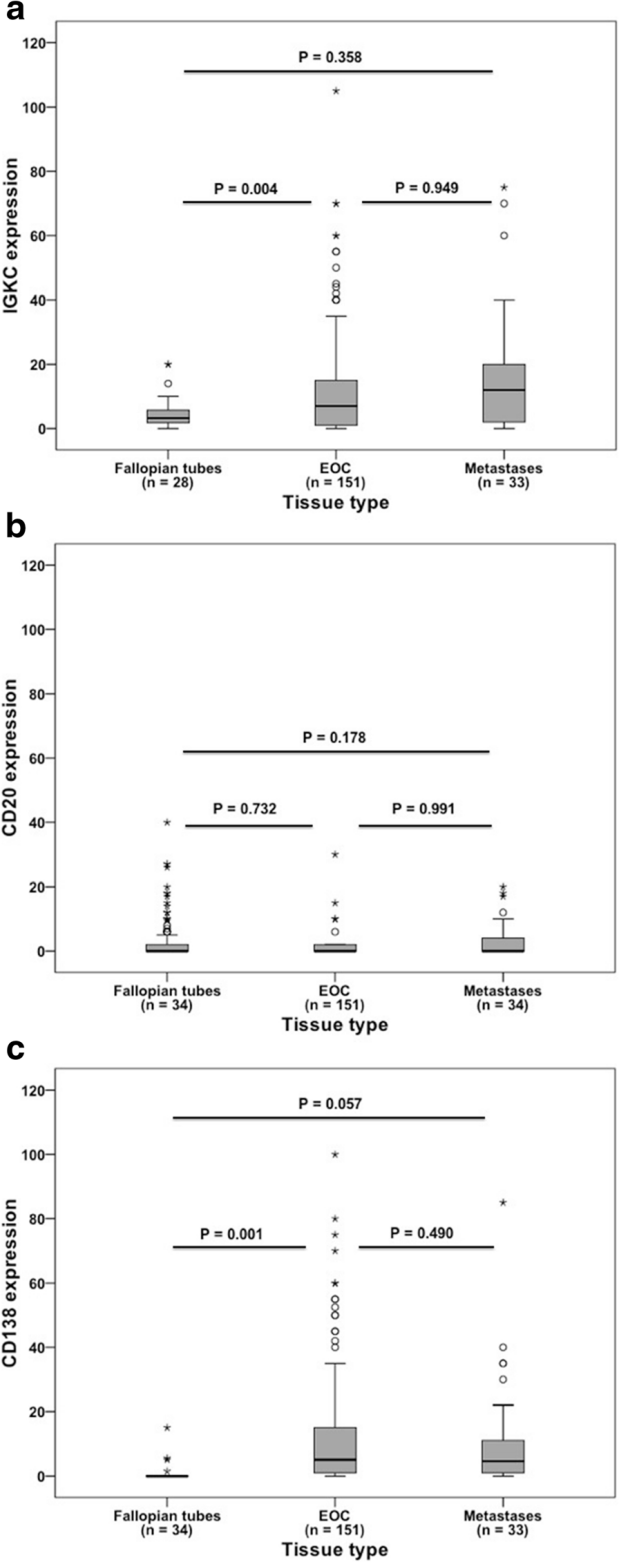
Distribution of IGKC, CD20 and CD138 expressions in fallopian tubes, primary tumours and metastases. Box plot visualising the staining distribution of (**a**) IGKC (**b**) CD20 and (**c**) CD138 in fallopian tubes, primary tumours and metastases. CD20 expression is described as core score, i.e. a multiplier of fraction in estimated percentage and intensity (0–2) of staining

The expression of the three biomarkers did not differ significantly between primary tumours and metastases, however; both IGKC and CD138 expression was found to be significantly higher in primary tumours than in fallopian tubes (*p* = 0.004 and *p* = 0.001, respectively) (Fig. 2).

### Interrelationship of IGKC, CD20 and CD138 expression and associations with clinicopathological factors

There were significant associations between expression of the three markers; IGKC and CD20 (*p* = 0.001), IGKC and CD138 (*p* = 0.0017) and CD20 and CD138 (*p* = 0.036) (Table 1). Furthermore, there was a significant inverse association between expression of CD20 and PIGR (*p* = 0.039). Both CD20 and CD138 expression were associated with high-grade tumours (*p* = 0.032 and *p* = 0.030, respectively), whereas no significant association was found between IGKC expression and differentiation grade. None of the three investigative biomarkers correlated significantly with age or clinical stage. Only IGKC expression was found to differ significantly between histological subtypes, with higher expression in clear cell type tumours (*p* = 0.026) (Table 2). Finally, higher CD20 expression was more often present in KRAS wild-type tumours (*p* = 0.027).

**Table 1:** Interrelationship between IGKC, CD20 and CD138 expression in primary tumours

Entire cohort					Serous carcinomas			
	IGKC	CD20	CD138	PIGR	IGKC	CD20	CD138	PIGR
IGKC								
*R*		0.257**	0.196*	0.008		0.237*	0.113	−0.027
*p*		0.001	0.017	0.923		0.025	0.297	0.805
*n*		151	148	150		89	87	89
CD20								
*R*	0.257**		0.171*	−0.167*	0.237*		0.126	−0.083
*p*	0.001		0.036	0.039	0.025		0.244	0.439
*n*	151		151	153	89		88	90
CD138								
*R*	0.196*	0.171*		−0,087	0.113	0.126		0.100
*p*	0.017	0.036		0.292	0.297	0.244		0.356
*n*	148	151		150	87	88		88

*R* = Spearman’s correlation coefficient, *p* = p-value, *n* = number of cases available for analysis. IGKC = immunoglobulin kappa c; PIGR = polymeric immunoglobulin receptor. *significance at 5 % level, ** significance at 1 % level. The analysis are based on cytoplasmic score (multipliers of staining intensity and fraction) for PIGR and core score for IGKC, CD20 and CD138

**Table 2:** Associations between IGKC, CD20 and CD138 expression and clinicopathological and investigative factors

Factor	IGKC expression mean/median (range)	CD20 expression mean/median (range)	CD138 expression mean/median (range)
Age			
*p*	*0.886*	*0.879*	*0.067*
≤Median	12.34/ 8.75 (0.00–70.00)	3.16// 0.00 (0.00–40.00)	9.24/ 5.00 (0.00–60.00)
>Median	13.25/7.00 (0.00–105.00)	2.43/0.00 (0.00–27.00)	16.53/6.00 (0.00–100.00)
Histological subtype			
*p*	*0.026*	*0.099*	*0.088*
Serous	12.55/7.00 (0.00–1 05.00)	3.32/0.25 (0.00–40.00)	12.15/5.25 (0.00–75.00)
Endometroid	8.92/4.50 (0.00–60.00)	2.24/0.00 (0.00–27.00)	13.76/5.00 (0.00–80.00)
Mucinous	10.40/7.50 (0.00–32.00)	1.08/0.00 (0.00–10.00)	8.25/1.25 (0.00–70.00)
Others	27.81/20.00 (0.00–70.00)	2.05/0.00 (0.00–18.00)	15.89/11.00 (0.00–100.00)
Differentiation grade			
*p*	*0.403*	*0.032*	*0.030*
Low	13.50/7.00 (0.00–105.00)	3.00/0.00 (0.00–27.00)	13.47/6.75 (0.00–100.00)
High	11.14/7.00 (0.00–70.00)	2.35/0.00 (0.00–40.00)	11.69/2.50 (0.00–80.00)
Clinical stage			
*p*	*0.443*	*0.880*	*0.388*
I	14.92/8.25 (0.00–70.00)	3.17/0.00 (0.00–20.00)	12.38/5.00 (0.00–80.00)
II	9.05/7.00 (0.00–32.50)	3.72/0.00 (0.00–27.00)	6.33/5.00 (0.00–30.00)
III	12.64/7.25 (0.00–70.00)	2.38/0.00 (0.00–40.00)	14.64/7.00 (0.00–70.00)
IV	15.93/7.25 (0.00–105.00)	1.81/0.00 (0.00–17.00)	16.83/5.00 (0.00–100.00)
KRAS mutation status			
p	0.076	0.027	0.135
*Wild-type*	13.26/7.50 (0.00–105.00)	3.08/0.00 (0.00–40.00)	13.29/6.00 (0.00–100.00)
Mutated	9.50/1.00 (0.00–70.00)	0.68/0.00 (0.00–10.00)	10.53/2.50 (0.00–70.00)

IGKC = immunoglobulin kappa C. The analysis of biomarker expression was based on a multiplier of staining intensity and fraction of immune cell staining

### Prognostic significance of IGKC, CD20 and CD138 expression

For IGKC expression, CRT analysis established an optimal cut-off point at CS ≤ 28.5, which was used to stratify cases into groups of low (CS ≤ 28.5, n = 128) and high expression (CS > 28.5, n = 23), respectively (Additional file 1). Using the same method, cases were divided into groups of low (CS ≤ 0.75, *n* = 93) and high CD20 expression (CS >0.75, *n* = 61). Neither IGKC nor CD20 expression showed any significant association with OS or OCSS in Kaplan-Meier analysis (data not shown).

**Fig. 3:**
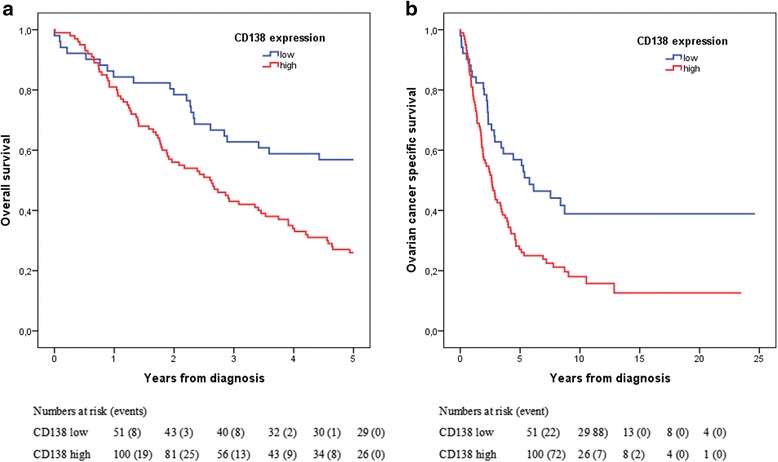
Kaplan-Meier estimates of ovarian cancer specific and overall survival in all patients according to CD138 expression. Kaplan Meier analysis of (**a**) overall survival and (**b**) ovarian cancer specific survival in strata of low and high CD138 expression. The categories of staining were determined by classification and regression tree analysis based on the core score (CS), whereby low expression = CS ≤ 2.25 and high expression = CS > 2.25

Similarly, immune cell-specific CD138 expression was dichotomized into groups of low (CS ≤ 2.25, *n* = 51) and high expression (CS >2.25, *n* = 100). Kaplan-Meier analysis revealed a significant association between high immune cell specific expression of CD138 and poor OS (*p* = 0.001) as well as OCSS (*p* = 0.002) (Fig. 3). These associations were also present in the subgroup of serous carcinoma (data not shown). Univariable Cox regression analysis confirmed the relationship between high CD138 expression and a decreased OS (HR = 2.20; 95 % CI 1.34-3.55; *p* = 0.001) as well as OCSS (HR = 1.95; 95 % CI 1.28–2.98; *p*–0.002). These associations remained significant in multivariable analysis, adjusted for age, stage and grade for OS (HR = 2.28; 95 % CI 1.39–3.75; *p* = 0.001) and OCSS (HR = 1.97; 95 % CI 1.27–3.05; *p* = 0.002).

We also examined the prognostic impact of a combined variable of CD138 and CD20 expression. As shown in Additional file 2, cases with high expression of both CD138 and CD20 had a significantly poorer OS in Kaplan-Meier analysis (*p* = 0.001), as compared to reference cases with CD138 low/CD20 low immune cell-specific expression, as did CD138 high/CD20 low cases (*p* = 0.003). Univariable Cox regression analysis confirmed this relationship (HR = 2.86; 95 % CI 1.50–5.44; *p* = 0.001 for CD138 high/CD20 high and HR = 2.51; 95 % CI 1.34–4.70; *p* = 0.004 for CD138 high/CD20 low cases). However, these associations did not remain significant in adjusted analysis (data not shown). OCSS showed similar trends to OS between the reference group, CD138 high/CD20 high, and CD138 high/CD20 low (data not shown).

Tumour-specific CD138 expression did not correlate significantly with prognosis (data not shown).

## Discussion

Multiple studies have previously described associations between infiltrating immune cells, prognosis and treatment response in cancer; nonetheless, the clinical relevance has most often been attributed to the T-cell linage [24]. In EOC, the infiltration of T-lymphocytes has been associated with markedly prolonged survival in multiple studies [15, 25–29], while the prognostic impact of tumour-infiltrating B cell and plasma cell markers is more unexplored. This study comprehensively analysed the prognostic significance of B-cell and plasma cell markers in EOC.

To the best of our knowledge, this is the first report to describe the prognostic impact of immune cell-specific CD138 expression in EOC. Interestingly, we found high immune cell-specific CD138 expression to be an independent prognostic marker for shorter survival in EOC. This is in line with previous findings in breast cancer [30], but in contrast with the study in non-small cell lung cancer by Lohr *et al.*, wherein high immune cell-specific CD138 expression was found to correlate with improved patient outcome [9].

Chronic inflammation is known to be a major factor in the development and progression of EOC, incurred by e.g. ovulation, [31] and previous findings demonstrate local activation of B-cells to cause neoplasms [32]. Mohammed *et al.* lay forward a plausible hypothesis for the prognostic impact of CD138+ TILs based on these facts, whereby a large CD138+ subpopulation is suggested to suppress T-cell response or to promote tumour progression by nurturing an inflammatory microenvironment [30].

Further, B-cells are able to attenuate chemotherapy response in squamous cell carcinoma [33] by fostering angiogenesis and inhibiting T-cell response. In a mouse model, B-cells have been shown to antagonize the tumour suppressing effects of chemotherapy and T-cells [34]. As the vast majority of the patients included in the present study received chemotherapy, the reduced survival rates of patients with tumours displaying a high CD138+ TIL count may be explained by the interference of B-cells and plasma cells in chemotherapy response. Aforementioned findings and the findings of the present study highlight the janus-faced role of the humoral immune system and the potential of using B-cells as therapeutic targets in cancer treatment.

CD138 has, in contrast to IGKC, a broader staining profile with reactivity in tumour cells and tumoural stroma besides plasma cells. Several studies have reported high stromal- or tumour-specific CD138 to be associated with poor patient outcome in various types of cancer, including EOC [35–37] and Rousseau *et al.* described CD138 as a promising new target for immunotherapy in metastatic breast cancer [38]. This could be explained by the biological functions of CD138, which have been shown to affect several steps in tumour progression and to facilitate metastasis [39] and increased chemotherapy resistance [40]. On the other hand, Kusumoto *et* al. reported loss of epithelial CD138 to correlate with improved prognosis in EOC [12]. In the present study, however, stromal expression was not accounted for and tumour-specific CD138 expression was not prognostic. Moreover CD138 expression was found be significantly higher in KRAS wild-type tumours. This association is well in line with the previously demonstrated association of KRAS wild-type with high tumour grade and reduced survival in the herein investigated cohort [23].

In addition, we found a significant correlation between expression of CD138 and IGKC, consistent with previous research indicating also the reliability of the immunohistochemical markers for the plasma cells lineage [9]. Although IGKC should be a more convenient marker for biomarker studies related to plasma cells [41], this study found no significant prognostic impact of IGKC expression. These findings are analogous with what Schmidt and colleagues previously demonstrated at the gene expression level in 426 cases of EOC [10]. In non-small cell lung cancer the prognostic value of IGKC and CD138 was found to be similar [9] but although IGKC is a more specific marker for plasma cells, the prognostic value of IGKC and CD138 may differ in different types of cancer, depending on the microenvironment and possibly also in relation to adjuvant chemotherapy. Further studies are warranted to elucidate the prognostic and potential predictive value of CD138 and IGKC in EOC.

In high-grade serous EOC, high infiltration of CD20+ B cells has been associated with prolonged survival [15], an observation that was not confirmed in our study. Nielsen *et al.* demonstrated CD20+ and CD8+ TILs to work cooperatively to mediate anti-tumour immunity, leading to markedly prolonged patient survival [14]. Further, Kroeger et al. showed that, in high-grade serous EOC, plasma cells were associated with infiltration of other lymphocyte population such as CD8+ cells and an active cytotoxic anti-tumour response [42]. In addition, the study demonstrated that the prognostic impact of CD8+ cells was only evident in tumours with infiltration of other types of lymphocyte populations such as CD20+ cells and plasma cells. The present study only examined the expression and prognostic impact of B cells and plasma cells, but it would be also of interest to examine the interrelationship and prognostic impact of various subsets of T-cells and B-cells in future studies. Of note, in the present study, we demonstrated high B-cell and plasma cell infiltration in combination to be an indicator of poor prognosis, although not independent of other prognostic factors.

It can be surmised that CD20+ TILs primarily function as antigen presenting cells and thus need cooperation with the cellular immune response to effect tumour progression. CD20 expression was found to correlate significantly with high tumour grade. Furthermore, CD20 expression was revealed to have an inverse relationship with tumour-specific expression of PIGR, a receptor that binds polymeric immunoglobulin molecules at the surface of epithelial cells. High expression of PIGR has been demonstrated to correlate with an improved patient outcome in several cancer forms including EOC [16, 43–46] and, speculatively, this may be attributed to a negative regulatory function of PIGR on B-cell TILs, or vice versa.

## Conclusions

In conclusion, we have analysed several markers of the humoral immune response in EOC and identified immunological subgroups that independently correlate with clinical outcome. This results from this study supplement and extend the current knowledge on the immune landscape in EOC, and may thus provide further information to enable the development of immune modulatory treatment options.

### Ethics approval and consent to participate

Ethical permission for the study was approved by the Ethics Committee of Lund University (Ref 445/2007). All patients gave written consent.

### Consent for publication

Not applicable.

### Availability of data and material

All dataset on which the conclusions are based upon are deposited and presented in the article and additional files.

## Additional files

Additional file 1: Classification and regression tree analysis. Results from classification and regression tree analysis for expression of (A) IGKC, (B) CD20 and (C) CD138 in relation to overall survival. (TIF 1117 kb) Additional file 2: Kaplan-Meier estimates of overall survival in all patients according to a combined variable of CD20 and CD138 expression. Kaplan-Meier analysis of overall survival in strata according to combinations of high or low immune-cell specific expression of CD20 and CD138, respectively. (TIFF 18229 kb)

## Abbreviations

CRT: classification regression tree
CS: core score
EOC: epithelial ovarian cancer
HR: hazard ratio
IHC: immunohistochemistry
IgA: immunoglobulin A
IGKC: immunoglobulin kappa c
MDCS: Malmö Diet and Cancer study
MPP: Malmö Preventive Project
OCSS: ovarian cancer specific survival
OS: overall survival
PIGR: polymeric immunoglobulin receptor
TILs: tumour infiltrating lymphocytes
TMA: tissue microarray

## References

[1] Hanahan D, Weinberg RA (2011). Hallmarks of cancer: the next generation. Cell.

[2] Ostrand-Rosenberg S (2008). Immune surveillance: a balance between protumor and antitumor immunity. Curr Opin Genet Dev.

[3] Grivennikov SI, Greten FR, Karin M (2010). Immunity, inflammation, and cancer. Cell.

[4] Lin WW, Karin M (2007). A cytokine-mediated link between innate immunity, inflammation, and cancer. J Clin Invest.

[5] Lynch TJ, Bondarenko I, Luft A, Serwatowski P, Barlesi F, Chacko R (2012). Ipilimumab in combination with paclitaxel and carboplatin as first-line treatment in stage IIIB/IV non-small-cell lung cancer: results from a randomized, double-blind, multicenter phase II study. J Clin Oncol.

[6] Robert C, Thomas L, Bondarenko I, O'Day S, Weber J, Garbe C (2011). Ipilimumab plus dacarbazine for previously untreated metastatic melanoma. N Engl J Med.

[7] Fridman WH, Pages F, Sautes-Fridman C, Galon J (2012). The immune contexture in human tumours: impact on clinical outcome. Nat Rev Cancer.

[8] Schmidt M, Bohm D, von Torne C, Steiner E, Puhl A, Pilch H (2008). The humoral immune system has a key prognostic impact in node-negative breast cancer. Cancer Res.

[9] Lohr M, Edlund K, Botling J, Hammad S, Hellwig B, Othman A (2013). The prognostic relevance of tumour-infiltrating plasma cells and immunoglobulin kappa C indicates an important role of the humoral immune response in non-small cell lung cancer. Cancer Lett.

[10] Schmidt M, Hellwig B, Hammad S, Othman A, Lohr M, Chen Z (2012). A comprehensive analysis of human gene expression profiles identifies stromal immunoglobulin kappa C as a compatible prognostic marker in human solid tumors. Clin Cancer Res.

[11] Matsuzaki H, Kobayashi H, Yagyu T, Wakahara K, Kondo T, Kurita N (2005). Reduced syndecan-1 expression stimulates heparin-binding growth factor-mediated invasion in ovarian cancer cells in a urokinase-independent mechanism. Oncol Rep.

[12] Kusumoto T, Kodama J, Seki N, Nakamura K, Hongo A, Hiramatsu Y (2010). Clinical significance of syndecan-1 and versican expression in human epithelial ovarian cancer. Oncol Rep.

[13] Davies EJ, Blackhall FH, Shanks JH, David G, McGown AT, Swindell R (2004). Distribution and clinical significance of heparan sulfate proteoglycans in ovarian cancer. Clin Cancer Res.

[14] Nielsen JS, Sahota RA, Milne K, Kost SE, Nesslinger NJ, Watson PH (2012). CD20+ tumor-infiltrating lymphocytes have an atypical CD27- memory phenotype and together with CD8+ T cells promote favorable prognosis in ovarian cancer. Clin Cancer Res.

[15] Milne K, Kobel M, Kalloger SE, Barnes RO, Gao D, Gilks CB (2009). Systematic analysis of immune infiltrates in high-grade serous ovarian cancer reveals CD20, FoxP3 and TIA-1 as positive prognostic factors. PLoS One.

[16] Berntsson J, Lundgren S, Nodin B, Uhlen M, Gaber A, Jirstrom K (2014). Expression and prognostic significance of the polymeric immunoglobulin receptor in epithelial ovarian cancer. J Ovarian Res.

[17] Berglund G, Elmstahl S, Janzon L, Larsson SA (1993). The Malmo Diet and Cancer Study. Design and feasibility. J Intern Med.

[18] Nilsson PM, Nilsson JA, Berglund G (2006). Population-attributable risk of coronary heart disease risk factors during long-term follow-up: the Malmo Preventive Project. J Intern Med.

[19] Nodin B, Hedner C, Uhlen M, Jirstrom K (2012). Expression of the global regulator SATB1 is an independent factor of poor prognosis in high grade epithelial ovarian cancer. J Ovarian Res.

[20] Ehlen A, Brennan DJ, Nodin B, O'Connor DP, Eberhard J, Alvarado-Kristensson M (2010). Expression of the RNA-binding protein RBM3 is associated with a favourable prognosis and cisplatin sensitivity in epithelial ovarian cancer. J Transl Med.

[21] Ehlen A, Nodin B, Rexhepaj E, Brandstedt J, Uhlen M, Alvarado-Kristensson M (2011). RBM3-regulated genes promote DNA integrity and affect clinical outcome in epithelial ovarian cancer. Transl Oncol.

[22] Nodin B, Fridberg M, Uhlen M, Jirstrom K (2012). Discovery of dachshund 2 protein as a novel biomarker of poor prognosis in epithelial ovarian cancer. J Ovarian Res.

[23] Nodin B, Zendehrokh N, Sundstrom M, Jirstrom K (2013). Clinicopathological correlates and prognostic significance of KRAS mutation status in a pooled prospective cohort of epithelial ovarian cancer. Diagn Pathol.

[24] Mantovani A, Romero P, Palucka AK, Marincola FM (2008). Tumour immunity: effector response to tumour and role of the microenvironment. Lancet.

[25] Nelson BH (2008). The impact of T-cell immunity on ovarian cancer outcomes. Immunol Rev.

[26] Zhang L, Conejo-Garcia JR, Katsaros D, Gimotty PA, Massobrio M, Regnani G (2003). Intratumoral T cells, recurrence, and survival in epithelial ovarian cancer. N Engl J Med.

[27] Hwang WT, Adams SF, Tahirovic E, Hagemann IS, Coukos G (2012). Prognostic significance of tumor-infiltrating T cells in ovarian cancer: a meta-analysis. Gynecol Oncol.

[28] Hamanishi J, Mandai M, Iwasaki M, Okazaki T, Tanaka Y, Yamaguchi K (2007). Programmed cell death 1 ligand 1 and tumor-infiltrating CD8+ T lymphocytes are prognostic factors of human ovarian cancer. Proc Natl Acad Sci U S A.

[29] Sato E, Olson SH, Ahn J, Bundy B, Nishikawa H, Qian F (2005). Intraepithelial CD8+ tumor-infiltrating lymphocytes and a high CD8+/regulatory T cell ratio are associated with favorable prognosis in ovarian cancer. Proc Natl Acad Sci U S A.

[30] Mohammed ZM, Going JJ, Edwards J, Elsberger B, McMillan DC (2013). The relationship between lymphocyte subsets and clinico-pathological determinants of survival in patients with primary operable invasive ductal breast cancer. Br J Cancer.

[31] Ness RB, Cottreau C (1999). Possible role of ovarian epithelial inflammation in ovarian cancer. J Natl Cancer Inst.

[32] DeNardo DG, Coussens LM (2007). Inflammation and breast cancer. Balancing immune response: crosstalk between adaptive and innate immune cells during breast cancer progression. Breast Cancer Res.

[33] Affara NI, Ruffell B, Medler TR, Gunderson AJ, Johansson M, Bornstein S (2014). B cells regulate macrophage phenotype and response to chemotherapy in squamous carcinomas. Cancer Cell.

[34] Chapoval AI, Fuller JA, Kremlev SG, Kamdar SJ, Evans R (1998). Combination chemotherapy and IL-15 administration induce permanent tumor regression in a mouse lung tumor model: NK and T cell-mediated effects antagonized by B cells. J Immunol.

[35] Nguyen TL, Grizzle WE, Zhang K, Hameed O, Siegal GP, Wei S (2013). Syndecan-1 overexpression is associated with nonluminal subtypes and poor prognosis in advanced breast cancer. Am J Clin Pathol.

[36] Juuti A, Nordling S, Lundin J, Louhimo J, Haglund C (2005). Syndecan-1 expression--a novel prognostic marker in pancreatic cancer. Oncology.

[37] Bodoor K, Matalka I, Hayajneh R, Haddad Y, Gharaibeh W (2012). Evaluation of BCL-6, CD10, CD138 and MUM-1 expression in diffuse large B-cell lymphoma patients: CD138 is a marker of poor prognosis. Asian Pac J Cancer Prev.

[38] Rousseau C, Ruellan AL, Bernardeau K, Kraeber-Bodere F, Gouard S, Loussouarn D (2011). Syndecan-1 antigen, a promising new target for triple-negative breast cancer immuno-PET and radioimmunotherapy. A preclinical study on MDA-MB-468 xenograft tumors. EJNMMI Res.

[39] Blackhall FH, Merry CL, Davies EJ, Jayson GC (2001). Heparan sulfate proteoglycans and cancer. Br J Cancer.

[40] Wang X, Zuo D, Chen Y, Li W, Liu R, He Y (2014). Shed Syndecan-1 is involved in chemotherapy resistance via the EGFR pathway in colorectal cancer. Br J Cancer.

[41] Schmidt M, Micke P, Gehrmann M, Hengstler JG (2012). Immunoglobulin kappa chain as an immunologic biomarker of prognosis and chemotherapy response in solid tumors. Oncoimmunology.

[42] Kroeger DR, Milne K, Nelson BH. Tumor infiltrating plasma cells are associated with tertiary lymphoid structures, cytolytic T cell responses, and superior prognosis in ovarian cancer. Clin Cancer Res. 2016 10.1158/1078-0432.CCR-15-2762 26763251

[43] Ai J, Tang Q, Wu Y, Xu Y, Feng T, Zhou R (2011). The role of polymeric immunoglobulin receptor in inflammation-induced tumor metastasis of human hepatocellular carcinoma. J Natl Cancer Inst.

[44] Rossel M, Billerey C, Bittard H, Ksiazek P, Alber D, Revillard JP (1991). Alterations in polymeric immunoglobulin receptor expression and secretory component levels in bladder carcinoma. Urol Res.

[45] Fristedt R, Gaber A, Hedner C, Nodin B, Uhlen M, Eberhard J (2014). Expression and prognostic significance of the polymeric immunoglobulin receptor in esophageal and gastric adenocarcinoma. J Transl Med.

[46] Fristedt R, Elebro J, Gaber A, Jonsson L, Heby M, Yudina Y (2014). Reduced expression of the polymeric immunoglobulin receptor in pancreatic and periampullary adenocarcinoma signifies tumour progression and poor prognosis. PLoS One.

