# Supplementary figures and images for: Prognostic impact of tumour-associated B cells and plasma cells in epithelial ovarian cancer
Source: J Ovarian Res. 2016 Apr 6;9:21. doi: 10.1186/s13048-016-0232-0 (PMC4822228; doi:10.1186/s13048-016-0232-0)

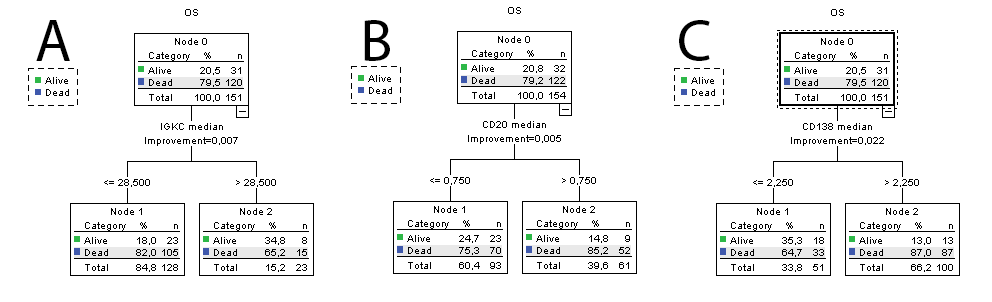

Supplement: Additional file 1: — Classification and regression tree analysis. Results from classification and regression tree analysis for expression of (A) IGKC, (B) CD20 and (C) CD138 in relation to overall survival. (TIF 1117 kb) [file 13048_2016_232_MOESM1_ESM.tif]

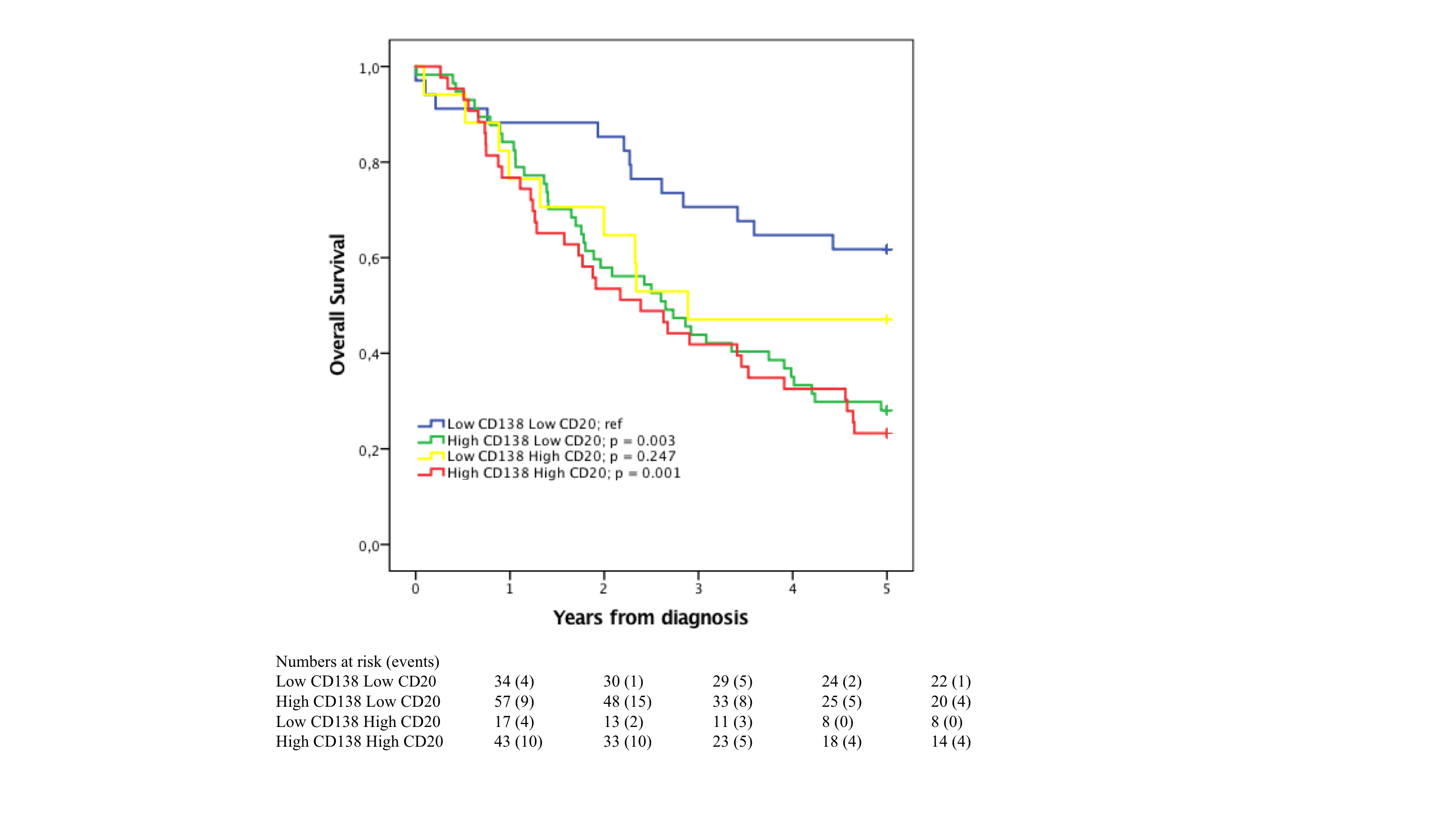

Supplement: Additional file 2: — Kaplan-Meier estimates of overall survival in all patients according to a combined variable of CD20 and CD138 expression. Kaplan-Meier analysis of overall survival in strata according to combinations of high or low immune-cell specific expression of CD20 and CD138, respectively. (TIFF 18229 kb) [file 13048_2016_232_MOESM2_ESM.tiff]
